# Effect of Different Lubricating Environment on the Tribological Performance of CNT Filled Glass Reinforced Polymer Composite

**DOI:** 10.3390/ma14112965

**Published:** 2021-05-31

**Authors:** Sandeep Agrawal, Nishant K. Singh, Rajeev Kumar Upadhyay, Gurminder Singh, Yashvir Singh, Sunpreet Singh, Catalin I. Pruncu

**Affiliations:** 1 Department of Mechanical Engineering, Hindustan College of Science and Technology, Farah, Mathura 281122, Uttar Pradesh, India; san197012@rediffmail.com (S.A.); nishant.singh78@gmail.com (N.K.S.); rajeevkrupadhyay@rediffmail.com (R.K.U.); 2 School of Mechanical and Materials Engineering, University College Dublin, Belfield, D04 V1W8 Dublin, Ireland; 3 Department of Mechanical Engineering, Graphic Era (Deemed to be University), Dehradun 248002, Uttarakhand, India; yashvirsingh21@gmail.com; 4 Mechanical Engineering, National University of Singapore, Singapore 117584, Singapore; snprt.singh@gmail.com; 5 Department of Mechanical Engineering, Imperial College London, Exhibition Rd., London SW7 2AZ, UK; 6 Design, Manufacturing & Engineering Management, University of Strathclyde, Glasgow G1 1XJ, UK

**Keywords:** carbon nanotubes, glass fiber reinforced polymer, friction, wear, sliding environment

## Abstract

In recent years, the engineering implications of carbon nanotubes (CNTs) have progressed enormously due to their versatile characteristics. In particular, the role of CNTs in improving the tribological performances of various engineering materials is well documented in the literature. In this work, an investigation has been conducted to study the tribological behaviour of CNTs filled with glass-reinforced polymer (GFRP) composites in dry sliding, oil-lubricated, and gaseous (argon) environments in comparison to unfilled GFRP composites. The tribological study has been conducted on hardened steel surfaces at different loading conditions. Further, the worn surfaces have been examined for a particular rate of wear. Field-emission scanning electron (FESEM) microscopy was used to observe wear behaviours. The results of this study explicitly demonstrate that adding CNTs to GFRP composites increases wear resistance while lowering friction coefficient in all sliding environments. This has also been due to the beneficial strengthening and self-lubrication properties caused by CNTs on GFRP composites, according to FESEM research.

## 1. Introduction

Because of their unique mechanical properties, low density, and wear and friction, glass fibre reinforced polymer (GFRP) composites have gained a lot of popularity as excellent and exciting ideas for engineering materials such as aviation brake discs, liners, boats, marine, automobile, and nozzles [1]. Since GFRP composites are used in many tribological applications, the tribological conduct of GFRP composites has indeed been studied [2,3]. The wear and friction coefficients for composite materials are higher and higher in inert gas environments than in air [4,5]. Agrawal et al. [6] discussed film formation using oil lubricant during wear testing of GFRP. The soft debris was obtained to be inserted among the fibres, which reduced the friction coefficient. The results were relatively better than the dry and argon gas environments. Mathew et al. [7] explored the effect of structure orientation of GFRP on tribological performance. The properties of each layer of fibres have shown a strong influence on the obtained wear values. Sathishkumar et al. [8] reviewed the different sliding conditions with various fibre orientations like random, woven mat and longitudinal with ceramics. However, it increases the brittle nature of the properties. Yousif et al. [9] used two related research methods, pin-on-disc and block-on-ring, to analyze the wear and friction behaviour of chopped strand mat glass fiber polyester composites under wet touch conditions with varying loads (30 N to 100 N). This experiment was done for parallel and anti-parallel fibre orientations. They inferred from the investigation that the presence of water raised the roughness value in both directions. Furthermore, anti-parallel orientation outperformed parallel orientation in terms of wear and frictional resistance. Moreover, under heavy load conditions and higher speeds, the wear rate is higher [10]. Prior research has shown that the fibre and matrix affect the tribological properties of GFRP composites [11,12]. All previous research indicates that the fracture toughness of GFRP composites needs to be improved to further boost their suitability for tribological applications [13,14,15]. 

Due to their exceptional properties, carbon nanotubes (CNTs) have evoked broad international research interest [16,17]. It is known that CNT-filled composites have very superior properties [18,19,20]. The addition of 1 wt.% of polymer composite CNTs can increase their strength and tensile matrix multiplication by up to 20% and 40%, respectively [21]. On the other hand, the CNT-filled FRP (fibre reinforced polymer) composite’s tribological features were comparatively less studied. The stuffing of 0.5% by wt. has already been given. The composite rate may be significantly reduced with ultra-high molar mass polyethylene CNTs, but the coefficient of friction can be increased. These findings were thought to be related partly to the composite’s higher shear resistance [22]. Enhanced friction and wear property of aluminium-based composites with CNTs of up to 4 wt. (%) has also been reported [23]. It was also assumed that the improved wear and friction attributes of the CNT-poly-tetra-fluoro-ethylene composite were due to the increased strength of the CNTs [24]. It was believed that the separate CNTs from the composites inhibited close contact with worn surfaces during sliding and therefore decreased both the coefficient of friction and the rate of wear.Venkatesan et al. [25] have analyzed the behaviour of hybrid composite wear (glass fibre-carbon nanotube). Findings show that increasing the concentration of CNT in glass fibre reinforced polymer composites reduces wear rate. Besides, results have indicated an improvement in COF by increasing the concentration of CNT in composites. Bastwros et al. [26] performed a tribological analysis of Al-CNT composites. The results revealed a substantial reduction in COF levels. The decrease in COF was attributed to an improvement in composites’ hardness and wear resistance due to the inclusion of CNT in composites. Gandhi et al. [27] explore the tribological conduct of hybrid composites combining polypropylene and carbon nanotube. The increase in load triggers an increase in wear. The rate of wear decreased as the concentration of CNTs increased. If there are higher proportions of CNT, a higher resistance to wear is observed. Besides, results also show that the rise in the concentration of CNT dramatically improves wear resistance. Mucha et al. [28] evaluate the impact of multi-walled carbon nanotubes on the wear behaviour of CNT added fibre composites. Findings show that the resistance to wear increases with the addition of CNT in the composite. Further, the results indicated the best wear resistance is obtained for fibre composites with a CNT concentration of between 0.25 and 0.5 wt.%. Saka et al. [29] explored composites’ friction and wear performance using various fillers, such as carbon nanotubes, graphite, and a mixture of these two. The findings of the tribological study indicated a decrease in COF and wear rate with the addition of filler materials. It also shows that carbon nanotubes as an additive in composites significantly improve tribological behaviour compared to other filler materials. Shaabania [30] studied the tribological activity of hybrid composites with a mixture of multi-walled carbon nanotubes and epoxy-polyamide. They further investigated the effect of various concentrations of multi-walled carbon nanotubes on composites’ friction and wear behaviour patterns. The results showed that the addition of multi-walled carbon nanotubes enhanced tribological characteristics. The higher tribological properties were 0.5 wt. (percentage) multi-walled carbon nanotubes.

The above literature review shows that even though much research has been done on the wear and friction of different CNT-filled composites, the process of wear and alteration of the friction coefficient and its causes have not yet been investigated and wholly understood. This work aims to take CNT reinforced/filled epoxy polymers and CNT-filled glass fiber reinforced polymers and composites as a case-by-case for a detailed review of the contribution of CNTs to the GFRP composite wear characteristics. CNTs were applied to the epoxy resin used as filler in GFRP composites to improve wear and friction properties. CNT filling was chosen because it has exclusive properties that make it a possible reinforcement candidate, and there was also some proof that it could reduce the coefficient of wear and friction. Several studies [21,22,23,24,25,26,27,28,29,30] have recently been performed on CNT-filled FRP composites to improve their properties. However, information on improvements in wear and friction properties by including carbon nanotubes is not thoroughly investigated or commonly available. This work provides the outcome of the investigation into the addition of carbon nanotube to the wear and friction attributes of GFRP composites, the sliding in and gas (argon) environments. The established results can be used for improving the GFRP tribological performance in regards of different applications.

## 2. Materials and Methods

### 2.1. Materials and Preparation of Specimen

The preparation process of GFRP was considered from the study conducted by Wang et al. [31]. The chemical vapour deposition (CVD) process (provided by Nano lab) with a 15 to 20 nm diameter and a size of 10 to 15 μm was obtained from the multi-walled CNTs utilized in this research. To combine properly with epoxy resin, the CNTs were ultrasonically mixed for 1 h in acetone with high purity (0.1 mg/mL) and for one hour after the introduction of epoxy resin. Separation of acetone is again carried out by heating this solution to 75 °C with continuous stirring and disintegrating in vacuum conditions at 50 °C for 24 h. Most of the solvent would have been extracted by this process. In this epoxy resin filled with CNT, glass fibres were reinforced (epoxy resin was L-12, and hardener was provided with K-6, and a new composite sample was prepared. E-glass was used to prepare this composite material using a woven roving. The woven glass fabric was built of 600 GSM, and the diameter of the E-glass fibre was 10–20 μm. Four different laminates have been prepared with a CNT content of 10 wt. Percentage (0, 2.5, 5 and 10%).

**Figure 1:**
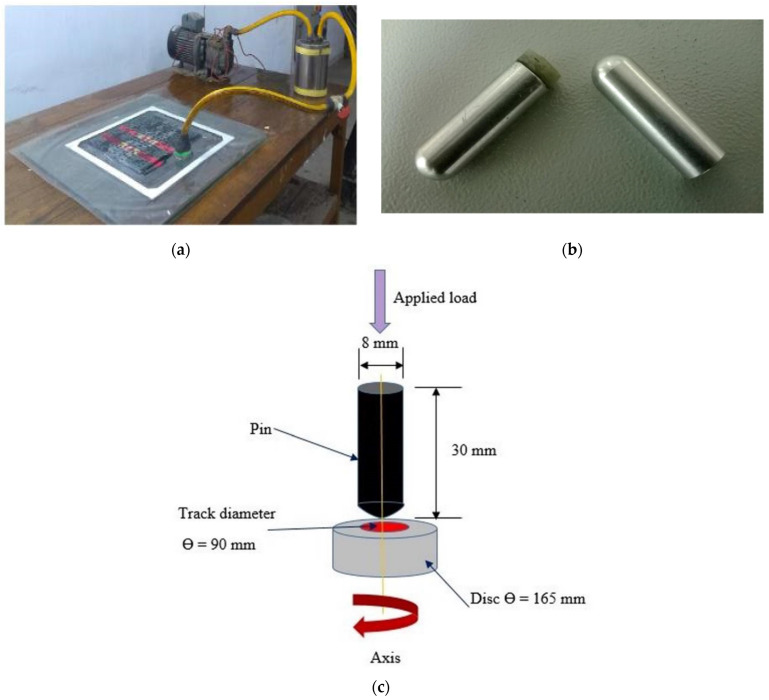
(**a**) Vacuum bagging setup used for fabrication of laminates and (**b**) fabricated pin samples (**c**) pin geometry.

The vacuum bagging process was used to make the composite plates of dimensions 270 mm × 320 mm × 4 mm using CNT-filled epoxy resin with glass fabric (see Figure 1a). A thickness of 4 mm was obtained by using glass fabric layers eight in numbers. The stacking sequence used was 0°/±45°/90°. The surface of the composite specimen was kept in touch with the disc made up of hardened steel having a 0° orientation of the fibre direction during sliding. The glass fabric was cut into the required number of layers, and each layer was placed in the predefined sequence having the warped face facing down. To provide easy gripping of layers during consolidation, the whole preparation process was carefully separated from contaminants like dust, grease, or other particulates that may cause trouble. At room temperature, these plates were cured for 48 h. To detect any unusual surface defects, the obtained plates were first examined by simple eye examination for any apparent defects. All plates were rendered free of any surface defect and delamination. Descriptions of the composites under investigation are given in Table 1. Sample pins were then made by sticking the CNT-filled GFRP composite having dimension (8 mm × 30 mm× 4 mm) with an adhesive having 8 mm-diameter, 30 mm-length and 4 mm-radius of aluminium pins (see Figure 1b). Pin construction/geometry is shown in the schematic image (see Figure 1c). The pin is making point contact with the disc when the loads are applied on the pin. The formation of wear on the track occurs in circular form during the contact of pin and disc because the disc rotates circularly with the help of a motor attached to it. The track diameter on the disc is adjusted with the help of the system available with the machine. Before using pin and disc, they are cleaned with ethanol. After each test, the disc is rubbed with 100 m to 1200 m emery paper to remove surface irregularities and cleaned with ethanol to make the surface look like a mirror. Before weighing on the precision balance machine, the pin is also cleaned with ethanol. After determining the weight of the pin, it is wrapped in aluminum foil and placed in a vacuum oven to prevent oxidation.

### 2.2. Testing Process

All of the tests were carried out on a wear tester manufactured by DUCOM in Bangalore (India). A pin-on-disc machine specifically designed for higher PV conditions, such as 200 N load and 10 m/s velocities. Table 2 illustrates the specifications of the pin-on-disc tribometer. The experimental operating conditions are mentioned in Table 3. The process parameters and their range have been identified through the literature review and pilot run test. Beyond the range, the influence of parameters is insignificant. According to the previous studies, maximum friction and wear occur at a lower velocity so, and only 500 rpm is selected for the analysis. The applied load is considered with a range 40 N to 120 N to analyze its effect on the surface. The working environment and material combination strongly influence the friction and wear behaviour of gas-lubricated systems. The adsorption of gas molecules and the chemical alteration of the contact surfaces significantly influence the behavior of each material. Based on the facts stated above, a comparison study was conducted utilizing three environmental conditions: dry, oil-lubricated, and gaseous (argon). Equation (1) was used to calculate the contact pressure concerning the contact area considered for the analysis.

(1) $$\mathrm{Contact}\;\mathrm{pressure}\;,\;p\;\left(\frac{N}{{\mathrm{mm}}^{2}}\right)=\frac{\mathrm{Applied}\;\mathrm{Load}\;\left(N\right)}{\mathrm{Area}\;\mathrm{of}\;\mathrm{contact}\;\left({\mathrm{mm}}^{2}\right)}$$

**Figure 2:**
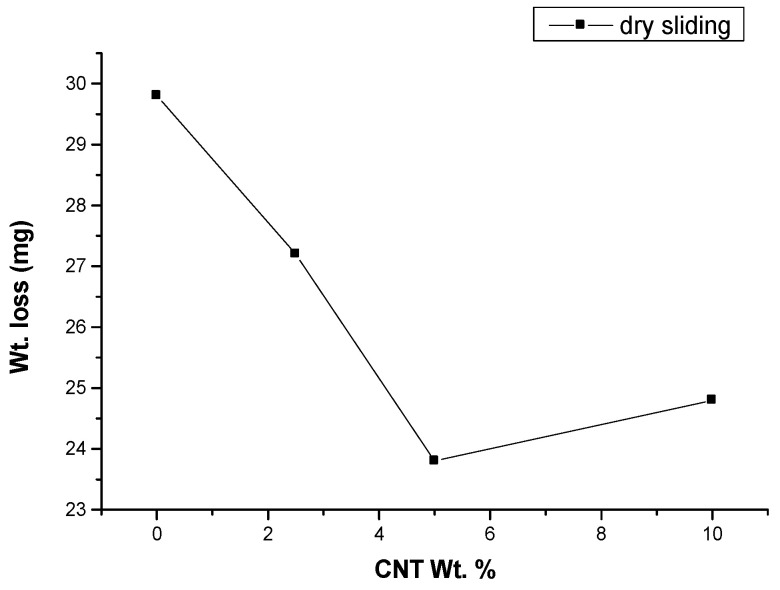
Wt. loss of CNT filled GFRP (wt.% 0, 2.5, 5 and 10) at 3.14 m/s, 120 N load and 2828 m sliding distance under dry sliding environment.

Initially, the sample pin’s (8 mm × 30 mm× 4 mm) face was smoothed by rubbing it against the side of a normal steel disk with a surface roughness of around 0.5–0.6 m, allowing the samples’ surfaces to connect seamlessly and without irregularities. In the experimental analysis, this smoothing of the touch face was often completed under similar loading conditions. A finer finishing disk made of EN 19 steel hardened to 55 HRC and having Ra values in the range of 0.1–0.2 m was then used to replace the disc. The primary weight of the pin was calculated after it had been cleaned with acetone and dried before the experiment began. It was smeared against the disk at two speeds: 2.15 m/s (500 rpm at 75 mm track diameter) and 2.95 m/s (500 rpm at 75 mm track diameter) (500 rpm at 90 mm track diameter). As a result, by sliding the pins for 500 s and 800 s, respectively, to obtain a significant amount of wear, a total distance of 1.608 km and 2.827 km was achieved. A precision balance of 0.01 mg was used to calculate the weight of the pin once more (after washing and drying the pin using the same method). To begin with, one of the composite samples has a different CNT weight percent. Under dry sliding conditions, the percentage was estimated at a sliding velocity of 3.14 m/s, a load of 120 N, and a sliding distance of 2.827 km. The study had a CNT content of 5%, which produced the best results, as seen in Figure 2. Each series of tests was repeated three times using the same procedure, and an appropriate weight loss value was calculated to determine the real wear rate value accurately. For loads of 40 N, 80 N, and 120 N, the experiments were carried out in the same way.

All experiments were performed in the adhesive wear analysis by rubbing samples under three environmental conditions of dry, oil-lubricated, and argon gas environments at specified experimental loads of 40 N, 80 N, and 120 N ambient temperature. A thermocouple was used to monitor the temperature rise with improvement in sliding. All these sets of experiments have been conducted, as mentioned above, for non-CNT-filled GFRP composites. Moreover, during experimentation, SAE 20 engine oil, with an apparent viscosity of 25–30 cSt at 50 cSt, has been used to lubricate the disc layer. Before beginning every trial, two drops of lubricating oil had been spread over the operating point. Then the oil was spilt at a fluid velocity of 0.02 mL/min during the experiment. Following the completion of the sliding process, the weight of the entire test specimen was recorded using an exact weighing balance machine after cleaning and curing with ethanol. Using a weighing balance machine, the weight was calculated by subtracting the initial and final weights of the pin during the test. The specific rate of wear was estimated by using the formula [31],

(2) $$\mathrm{SR}=\frac{\Delta M}{\rho Ld}$$

where ∆*M* is the weight difference (kg), *ρ* is the density (kg/mm^3^), *L* is the load (N) and *d* is the sliding distance (m).

## 3. Results and Discussion

### 3.1. Wear and Friction Attributes

**Figure 3:**
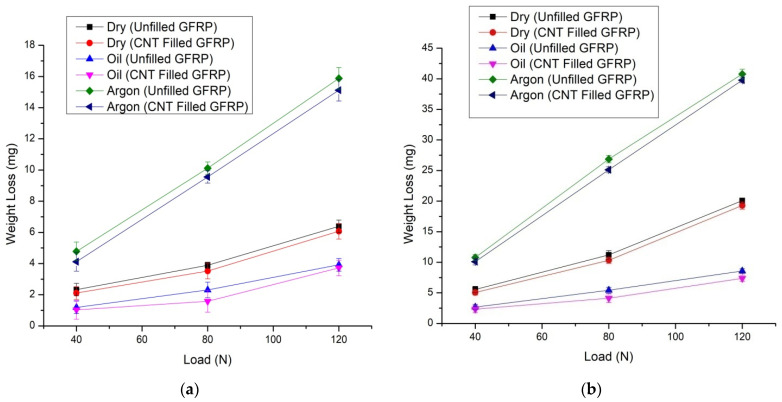
(**a**) Wt. Loss v/s Load for CNT-filled GFRP and unfilled GFRP composites under all three cases at 2.51 m/s and at 1508 m and (**b**) Wt. Loss v/s Load for CNT filled GFRP composites, during all the three cases at 3.14 m/s and for 2828 m.

The graph of weight loss vs. normal load for CNT-filled GFRP and unfilled GFRP composites can be seen in Figure 3a,b simultaneously. As a result, weight loss increased with an increase in load for all composites. The weight loss of the samples depends on the friction generated on the surface under different conditions when various loads are applied. The reduction in friction under oil lubricating conditions was observed in comparison to other environmental conditions. During oil lubrication on the surface, a better lubricant film is attained on the surface, leading to reduced friction, ultimately leading to less weight loss. The CNT also played a major role in reducing friction between the surfaces. CNTs have high mechanical strength and good self-lubricating properties that enhance the tribological characteristics of materials. So, the tendency of material resistant from wear increases with an increment in the percentage of CNT. The details of compressive strength and hardness of CNT were reported by Yan et al. [32] in their investigation. Under various sliding velocity, CNT filled GFRP composite sliding in oil-lubricated condition demonstrated the most significant wear resistance.

This behavior can be safely attributed to the presence of CNT in the body of CNT-filled GFRP composites, which act as an important means of preventing the large-scale disintegration of epoxy, leading to minor wear. This is obvious from the smaller size of the worn surface fragments observed in the FESEM study as discussed in Section 3.2. Many researchers [21,22,23] recognized the impact of CNT as an efficient filler. The glass fibres reinforce the composite, whereas the epoxy added to the CNT works together as binding agents to provide improved wear resistance. The wear of polymer composites occurs primarily due to velocity, load, and distance. Usually, the low depth of the softened layer leads to reduced wear. The CNT also has self-lubricating properties due to its structure, which is similar to graphite as reported by Guo et al. [33]. Figure 3a,b shows that losing weight improved with a higher sliding velocity/load value. The weight loss of all composite samples typically increases with the load applied at the constant velocity of sliding. The thickness of the softened layer of the epoxy matrix over the composite surface largely contributed to the hardness, making it easier for the softened layer to detach from the sample surface, resulting in weight loss to increase with higher loads, significantly due to the hardness and strength of the material. In both conditions, as shown in Figure 3a,b, weight loss in CNT filled GFRP was reduced by up to 12–18% when compared to unfilled GFRP in dry, oil, and argon environments.

#### 3.1.1. Specific Wear Rate

**Figure 4:**
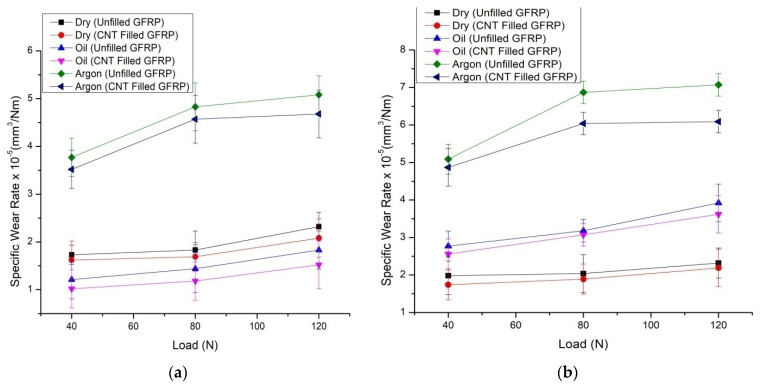
(**a**) Specific wear rate v/s Load for CNT filled GFRP and unfilled GFRP composites, during all the three environmental conditions at 2.51 m/s and for 1508 m, and (**b**) Specific wear rate v/s Load for CNT filled GFRP and unfilled GFRP composites, during all the three environmental conditions at 3.14 m/s and for 2828 m.

Figure 4a,b shows how the specific rate of wear on CNT filled GFRP and unfilled GFRP composites varies with load under all three environmental conditions. The introduction of CNT indicates a substantial decrease in the specific rate of wear in all three settings. This type of environmental deviation was given for unfilled GFRP composites [23], and a comparison of similar environmental performance between unfilled and CNT-filled GFRP results in the effect of CNT filling. Specific wear rates of unfilled GFRP and CNT-filled GFRP composites due to increasing normal loads in dry sliding, oil-lubricated, and argon environments are shown. It is noted that the individual rate of wear of CNT-filled GFRP composites has often shown a lower value compared to unfilled GFRP under all environmental conditions. This indicates beyond a reasonable doubt that the filling of CNTs has dramatically improved the wear resistance of unfilled GFRP composites during dry sliding and other lubricating conditions. Moreover, the ‘unique rate of wear’ of unfilled GFRP composites and CNT-filled GFRP composites sliding under argon environments has always been higher than the corresponding cases under ‘dry sliding environments’ accompanied by ‘oil-filled sliding environments’. This was on the predicted lines in the light of experiments reported by Agrawal et al. [7]. It is also seen from the statistics that the ‘unique rate of wear’ of all composites showed the same propensity with an increase in normal load, i.e., an increase in increasing load under all environmental conditions of dry sliding, oil-lubricated sliding, and argon environmental sliding. The ‘unique rate of wear’ of unfilled GFRP and CNT-filled GFRP composites increases both with a higher normal load, which is also on the predicted line, and in the same way as our earlier findings that the weight loss was relative to the normal load [24]. The separation of debris from the epoxy matrix in the case of CNT-filled GFRP composites is very different from that of unfilled GFRP, as shown by the re-filled GFRP composites.

Furthermore, caused by friction of the contact surfaces, the wear debris released has also played an important role. During the inert gas argon environmental state, the relative movement of the GFRP laminate to the steel disc does not cause the residue to stay in the peripheral portion of the fibres. The debris is isolated and flies away from the laminated area due to the relative motion combined with the gas flow, leading to a higher wear rate. As a result, new fibres encounter the mating component material, resulting in a higher friction value coefficient. A similar type of wear is also applicable in dry sliding of CNT-filled GFRP composites during sliding against hardened steel counter faces in all environments. In both conditions, as shown in Figure 4a,b, the specific wear rate of CNT filled GFRP was reduced by up to 6–13% when compared to unfilled GFRP in dry, oil, and argon environments.

#### 3.1.2. Coefficient of Friction

**Figure 5:**
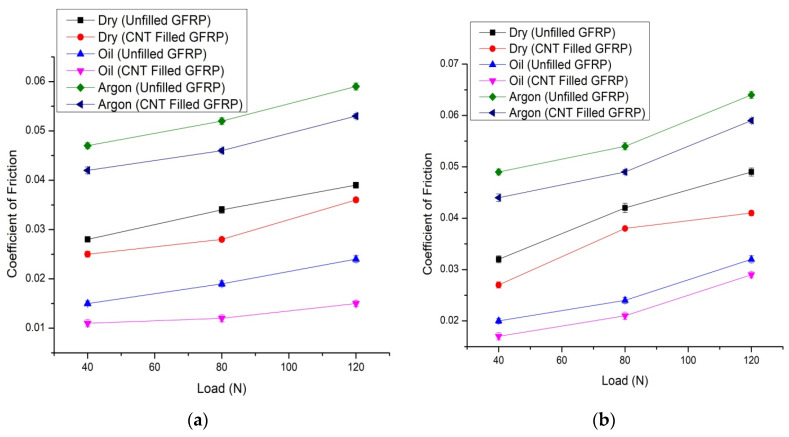
(**a**) Coefficient of friction v/s load for CNT filled GFRP and unfilled GFRP composites, during all the three cases at 2.51 m/s and for 1508 m, and (**b**) Coefficient of friction v/s normal load for CNT filled GFRP and unfilled GFRP composites, during all the three environmental conditions at 3.14 m/s and for 2828 m.

**Figure 6:**
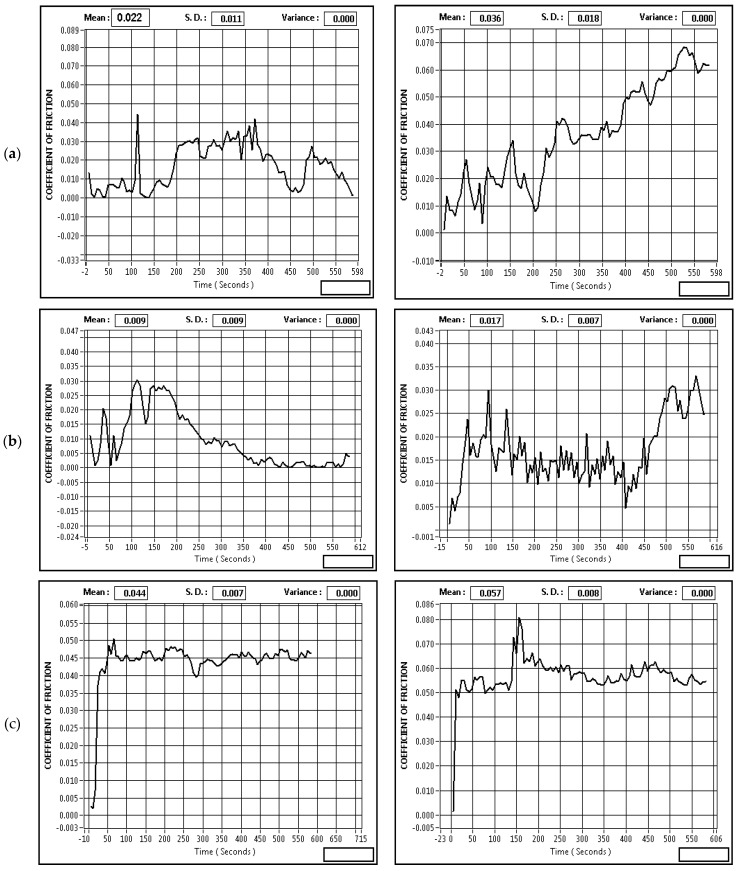
Coefficient of friction v/s time for CNT filled GFRP composites, during (**a**) dry sliding, (**b**) oil lubricated, and (**c**) argon sliding at 40 N, 120 N, at sliding speed 2.51 m/s and 1508 m.

The variation of the friction coefficient for CNT filled GFRP and unfilled GFRP composites at normal loads in dry sliding, oil hydrated, and argon scenarios with respect to the hardened steel counter-face can be seen in Figure 5a,b. This reveals that the friction coefficient for unfilled GFRP in dry sliding, oil-lubricated, and inert gas conditions is decreased due to the filling of CNTs. In the meantime, the coefficient of friction of unfilled GFRP and CNT-filled GFRP composites during oil hydration was always lower than the corresponding case during dry sliding, which shows that the oil is an efficient liquid that decreases the coefficient of friction for both composites, but the coefficient of friction is the lowest for CNT-filled GFRP during oil lubrication. From Figure 5, it can also be shown that the standard load affects the coefficient of friction of all composites during dry sliding, the argon environment, and the oil-lubricated state, delivering results in different ways. In dry sliding and inert gas, the friction coefficient for both composites increased at a larger scale with an improvement in normal load. However, on the other hand, under the oil hydrated scenario, the friction coefficient grew at a slower rate with an increase in load. At both conditions as mentioned in Figure 5a,b, the coefficient of friction was reduced upto 14–20% in CNT filled GFRP as compared to unfilled GFRP in dry, oil and argon environments. The coefficient of friction vs. time curves for all environmental conditions and the minimum and maximum loads for the lower value of the sliding speed are shown in Figure 6a–c.

CNT filling is an efficient tribological reinforcement for GFRP composites, as seen in the preceding discussion. The surface temperature of the composite increases during dry sliding because of increased heat generation due to increased friction, resulting in a rapid increase in adhesive friction. However, the temperature of the surface of CNT-filled GFRP composites increased less than that of unfilled GFRP composites. This was due to CNTs having a higher thermal conductivity than GFRP, which enhanced heat transfer caused by friction, and CNTs also acting as solid lubricants to reduce friction. All of this works together to reduce the friction coefficient of CNT-filled GFRP composites. Previous research has demonstrated that oil can be used as a lubricant and it reduces direct interaction when sliding the composite specimen face layer with the counter faces [23]. This resulting in a lower coefficient of friction in the event of a spill hydrated sliding than in the case of dry sliding due to the boundary layer effect of oil.

In addition, loss of the heat generated due to friction may be dissipated more easily, allowing the oil to cool down the composite specimen surface and counter face. Consequently, the friction coefficient for both the composites in the oil-lubricated condition is less than that in dry sliding. For the case of sliding wear of FRP composites against metallic counter faces, it is already known that friction resulting from adhesion is equal to the multiplication of the shear strength of the polymer and the actual contact area. With higher normal loads, the temperature between specimen and steel counter-face would usually increase because of heat generated due to friction, which results in two different effects on the coefficient of friction. Whereas on the one hand, shear strength of GFRP is reduced and as a result, the coefficient of friction for composites is also reduced; on the other hand, the GFRP elastic modulus is reduced at higher temperatures, thus resulting in an effective increase in the actual contact area and thus the coefficient of friction for GFRP is increased. Therefore, the final coefficient of friction for composites can be determined practically, combining the two competitive aspects. Finally, in this case, the coefficient of friction for unfilled GFRP composite and CNT filled GFRP composites is increased at a lower rate even at higher normal loads, under oil-lubricated condition due to the decrease in shear strength taking place. however, during dry sliding and argon environment, the coefficient of friction is raised at a higher rate at high normal loads because there is a rise in the real contact area. This study shows that the inert gas (argon) atmosphere results in a simple separation of the fibers from the polymer composite matrix during the wear process due to higher friction and thus optimum wear rate values. In addition, the soft epoxy resin structure matrix within composite layers during oil-lubricated sliding plays an essential part in lowering the coefficient of friction values. The lubricants are adsorbed on the steel disc surface, and the mild preliminary wear of the polymer often acts as a “lubricant shallow pot”. The bulbous fibres pick up these lubricating oil agents to prevent potential contact between the fibre and the steel and maintain efficient thin-film lubricating conditions. No such lubricant is available between the fibre layers in dry sliding conditions, as in oil-lubricated sliding. As a result, the fibres experience bending at the ends under the friction force resulting in fast shearing and, as a result, a higher rate of wear occurs.

### 3.2. FESEM Analysis

**Figure 7:**
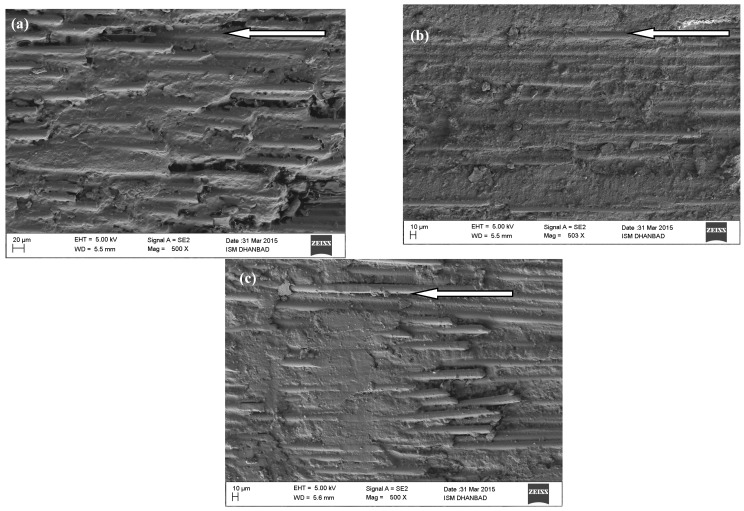
CNT filled GFRP composite worn out the surface for dry sliding at 3.14 m/s and for (**a**) 40 N, (**b**) 80 N and (**c**) 120 N.

The FESEM analyzed the surfaces of all worn samples to identify and better approach the wear process. As seen in Figure 7, the pictures are arranged to increase the rate of wear (WR). The arrow lines indicate the direction of the sliding. FESEM images of the worn-out surfaces of GFRP composites in dry sliding at normal loads of 40 N, 80 N, and 120 N. As shown in our previous paper [6], the worn surfaces of GFRP composite are extremely rough, which shows that the process of wear is due to adhesive and plough wear. Additionally, the worn paths of the GFRP composite specimen are parallel to the sliding direction and are relatively thick and deep. On the contrary, the adhesion and ploughing on the worn surface of CNT-filled GFRP composites are significantly decreased, as shown in Figure 7a–c. The worn surface is relatively smoother, indicating that the CNT filled GFRP composite epoxy matrix is not as easily separated when sliding against the steel face as in the unfilled case. This observation is also consistent with the enhanced wear resistance of CNT filled GFRP composites. Additionally, CNTs released from the composites during the sliding process may be entrapped in between the contact surfaces of the specimen and the steel face. These released CNTs work as a solid lubricant and help avoid direct contact, thereby reducing friction and wear rate. So it can be safely concluded that the filling of CNTs reduces the erosion and the adhesion of the GFRP composites in a dry sliding environment against the counter steel face. Consequently, the CNT filled GFRP composites give much improved results for the coefficient of friction and resistance to wear than those of the unfilled GFRP composite matrix.

**Figure 8:**
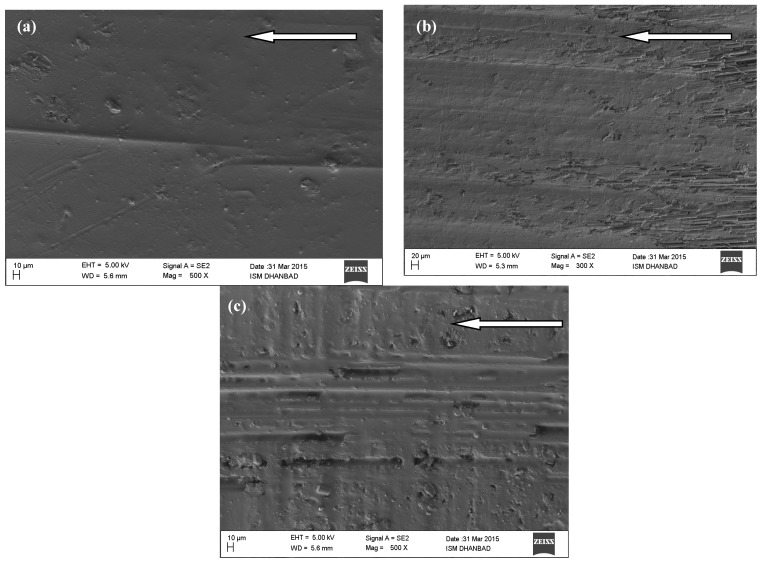
CNT filled GFRP composite worn out surface for oil-lubricated sliding at 3.14 m/s and for (**a**) 40 N, (**b**) 80 N and (**c**) 120 N.

The worn-out surfaces of GFRP composites in oil-lubricated conditions are seen in FESEM images at standard loads of 40 N, 80 N, and 120 N. The impact of natural load and slipping conditions on fiber damage in different mechanisms, as well as the restriction of deterioration in fiber–matrix bonding Furthermore, the fiber microcracks and microcuts, resulting in matrix disintegration and rubble, which is then thrown away from the floor, creating positive wear, or stuck in the matrix in various directions. It causes significant changes in the coefficient of friction, as seen in SEM spectra. As compared to the images of the CNT filled GFRP composites seen in Figure 8a–c, it is clear that the surface of the unfilled GFRP composite has worn off more badly under oil-lubricated conditions than the surface of the CNT filled GFRP composites under equal oil-lubricated sliding conditions. It is also worth noting that under oil-lubricated conditions, the worn off surface of an unfilled GFRP composite exhibits more crack regions and erosion.

**Figure 9:**
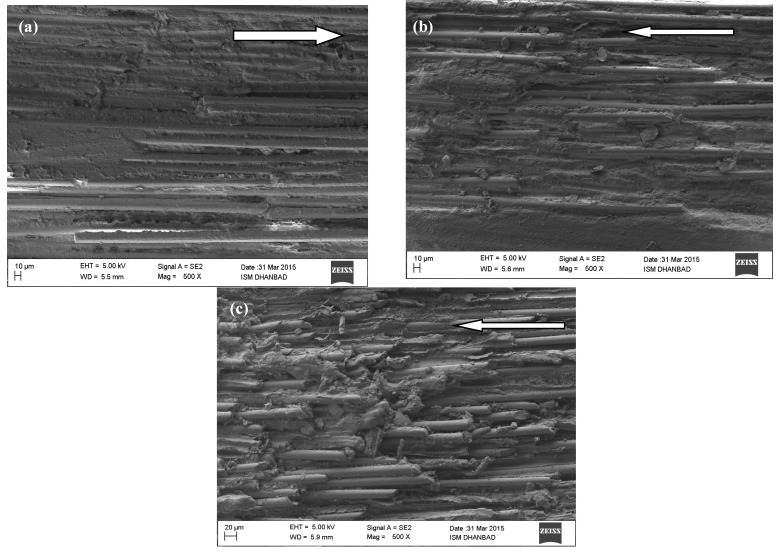
CNT filled GFRP composite worn out surface for argon environment sliding at 3.14 m/s and for (**a**) 40 N, (**b**) 80 N and (**c**) 120 N.

The FESEM picture of the worn-out surfaces of unfilled GFRP composites in inert argon gas, environmental conditions at normal loads of 40 N, 80 N, and 120 N are shown. By comparison with Figure 9a–c, the CNT images filled GFRP composite under similar conditions.

Once the load increased to 120 N, the wear loss grew substantially, resulting in an inertial fracture of the matrices, especially in the interfacial area. As a measure, the surface damage increased dramatically due to imprints created by the separating fibers. It has been observed clearly that CNT filled GFRP composites show more wear resistance than GFRP composites and much less erosion and plow marks because of their high crystalline and bending strength, which makes it highly difficult for the separation of fibres in argon environment sliding. In addition, argon may throw out the debris from the worn surface of the composite, which does not help in the transfer layer formation over the counter faces. Thus, the direct contact of the specimen with the sliding surfaces resulted in the greater wear of both the composites than the other two conditions.

## 4. Conclusions

In this study, the tribological properties of CNT-filled GFRP composites were investigated under dry sliding, oil-lubricated, and inert gas environmental conditions. The following conclusions were reached as a result of the findings:

- As the normal load increases from 40 N to 120 N, the weight loss, specific wear rate, and friction coefficient of GFRP composite are significantly decreased by filling with CNTs under all three environmental considerations of dry sliding, oil hydrated, and inert argon gas sliding. The effects are due to the distinct contributions of carbon nanotubes as a reinforcing effect in GFRP composites. For all three dry, tar, and argon environmental factors, weight loss, specific wear rate, and friction coefficient were decreased by up to 12–18%, 6–13%, and 13–20% in CNT filled GFRP relative to unfilled GFRP.
- Under dry sliding and inert argon gas sliding environmental conditions, the friction coefficient for both unfilled GFRP composites and CNT-filled GFRP composites improves faster with higher standard loads than oil-filled sliding. As compared to dry sliding and argon gas sliding, the friction coefficient for oil-filled sliding was 150–160% and 310–320%, respectively.
- In these three environmental conditions, the specific wear rate of CNT loaded GFRP composites increases as load values rise. FESEM photographs of all worn-out surfaces of unfilled GFRP composites and CNT loaded GFRP composites confirm and agree with the findings under all three environmental conditions at a typical load of 40 N, 80 N, and 120 N.

## Figures and Tables

**Table 1:** Variation of the temperature (°C) for CNT filled GFRP composite samples at different loads/sliding velocity and environmental conditions.

Environmental Condition	Velocity 2.51 m/s	Velocity 3.14 m/s
Dry Sliding		
40 N	41 °C	44 °C
80 N	44 °C	47 °C
120 N	47 °C	50 °C
Oil lubricated sliding		
40 N	36 °C	38 °C
80 N	39 °C	41 °C
120 N	41 °C	43 °C
Inert gas (Argon)		
40 N	45 °C	47 °C
80 N	48 °C	50 °C
120 N	51 °C	54 °C

**Table 2:** Specifications of the pin-on-disc tribometer.

Pin-on-Disc Tribometer	
Sliding speed, rpm	1–5000
Disc rotation speed, rpm	1–5000
Normal load, N	Maximum 200
Temperature, °C	Ambient-200
Disc size	160 mm dia. × 8 mm thick
Power	415 V, 15 Amps, 3 phase, 50 HZ

**Table 3:** Operating condition during test.

Operating Parameters	Value
Normal loads applied on the pin (N)	40, 80, 120
Contact pressure (N/mm^2^)	0.0091, 0.0181, 0.0272 at 75 mm and 0.0063, 0.0126, 0.0189 at 90 mm with respective loads
Track diameter used during test (mm)	75 and 90
Sliding speed, m/s	2.51 and 3.14
Sliding distance (km)	2.827
Size of the pin (mm)	(8 × 30 ×4)
Hardness of the pin (HRC)	50

## Abbreviations

CNTs	Carbon nanotubes
GFRP	Glass fiber reinforced polymer
COF	Coefficient of friction
SAE	Society of Automotive Engineers
CVD	Chemical vapor deposition
SR	Specific rate of wear
FRP	Fibre reinforced polymer
WR	Wear rate
FESEM	Field Emission Scanning Electron Microscope
